# Interspike Interval Based Filtering of Directional Selective Retinal Ganglion Cells Spike Trains

**DOI:** 10.1155/2012/918030

**Published:** 2012-08-02

**Authors:** Aurel Vasile Martiniuc, Alois Knoll

**Affiliations:** Computer Science Department VI, Technical University Munich, Boltzmannstraße 3, 85748 Garching, Germany

## Abstract

The information regarding visual stimulus is encoded in spike trains at the output of retina by retinal ganglion cells (RGCs). Among these, the directional selective cells (DSRGC) are signaling the direction of stimulus motion. DSRGCs' spike trains show accentuated periods of short interspike intervals (ISIs) framed by periods of isolated spikes. Here we use two types of visual stimulus, white noise and drifting bars, and show that short ISI spikes of DSRGCs spike trains are more often correlated to their preferred stimulus feature (that is, the direction of stimulus motion) and carry more information than longer ISI spikes. Firstly, our results show that correlation between stimulus and recorded neuronal response is best at short ISI spiking activity and decrease as ISI becomes larger. We then used grating bars stimulus and found that as ISI becomes shorter the directional selectivity is better and information rates are higher. Interestingly, for the less encountered type of DSRGC, known as ON-DSRGC, short ISI distribution and information rates revealed consistent differences when compared with the other directional selective cell type, the ON-OFF DSRGC. However, these findings suggest that ISI-based temporal filtering integrates a mechanism for visual information processing at the output of retina toward higher stages within early visual system.

## 1. Introduction

The information regarding visual stimulus is encapsulated initially in spike trains at the output of retina by retinal ganglion cells [1–4]. In some of mammals (though not general to mammals), the direction of stimulus motion is already signaled by the well-known directional selective retinal ganglion cells (DSRGCs) [5, 6]. They respond vigorously to the movement of stimulus at the preferred direction and are silent when stimulus movement is toward the opposite null direction [7]. In rabbit retina, one type of DSRGCs, known as the ON-OFF DSRGCs, has been already very well characterized [5, 6, 8–12]. They respond at the beginning and the end of an increasing or decreasing light stimulus and project to the dorsal lateral geniculate nucleus (LGN) and to the superior colliculus [11, 13].

Receptive fields (RFs) become progressively more sophisticated along the synaptic hierarchies from retina to cortex. However, for the LGN cells the center-surround RFs are similar to those of retinal afferents [14–16]. With this advantage in mind, together with the fact that the receptive field centers of LGN cells receive their main input from only one retinal ganglion cell (RGC) [17–19], the retinogeniculate synapse represents a major interest for studying the role of interspike interval-based mechanism for spike filtering and visual information processing [3, 20–23]. Already at the next stage within early visual system, neurons in layer 4 of primary visual cortex receive many more convergent inputs from LGN counterparts [16] and thus rely more on the interaction between different inputs than on the interspike intervals (ISIs) of individual inputs as part of the mechanism to reach the spike threshold [24].

 Many studies have shown that LGN cells seem to affect in an active manner the spike trains received from the retinal ganglion cells. It has been already demonstrated that LGN cells fire a lower number of spikes as compared to their retinal counterpart [18, 25, 26]. Consequently, not every retinal spike will evoke an action potential (AP) at the postsynaptic target in the LGN. The length of the ISIs of the retinal spike train represents an important factor in determining whether a retinal spike will evoke an AP at the LGN cell counterpart [18, 25–28].

Retinal spikes with preceding short ISIs have greater chances to induce APs at their postsynaptic target than “isolated” spikes. The efficacy to evoke APs at the postsynaptic target in LGN decreases considerably with increasing of retinal ISIs so that for retinal ISI larger than about 30 ms there is no detectable influence of ISI on the production of postsynaptic spikes [18, 25]. Furthermore, recent studies suggest that this temporal filter acting upon retinal spiking activity is correlated with visual stimulus, so that visual information regarding optimal stimulus features is preserved and transmitted further on at the postsynaptic target [21–23, 29].

To have a better intuition of how ISI-based temporal filter acts upon retinal spike trains, we analyzed previously recorded activity of different types of RGCs in response to two different types of stimuli (white noise and drifting grating bars) in rabbit retina [4].

Firstly, the results show that using white noise stimulus and reverse correlation analysis, we were able to notice that the strength of the correlation between stimulus and recorded neuronal response was at its maximum for shortest ISIs. We also found that correlation between stimulus and neural response decreases with increasing ISIs, and thus visual information varies with ISIs. This extremely important finding suggests that ISI-based temporal filter of retinal spike trains may influence the spike transfer at retinal postsynaptic target and serve to filter visual information from retina to higher stages. These findings are in concordance with previously reported in vivo results from cats [21].

We then went further and asked if ISI-based temporal filter remains robust for a different type of stimulus. Throughout the nervous system neurons respond selectively for different stimulus features (i.e., contrast, orientation, size). We focused on the optimal stimulus feature as being the stimulus direction of motion and analyzed the response of DSRGCs to the visual stimulus consisting in drifting grating bars, a commonly used stimulus to quantify the direction selectivity [4, 7, 30]. Our results from analysis of recorded activity of ON-OFF DSRGCs in response to drifting grating bars, presented at eight different directions, indicate that short ISIs were always tuned at preferred direction of stimulus movement and contribute to preserve the directional information. It is already known that optimal stimulus features induce higher firing rates and thus presumably short ISI spiking activity as well. To check if the tuning of short ISI is just strictly firing rate dependence and no other firing mechanism is involved, we built Poisson-like spike trains with similar tuning and firing rates as the recorded ON-OFF DSRGCs [31]. The discrepancy that we have noticed between the recorded cells and Poisson-like spike trains regarding the short ISI distribution and firing rates leads us to suggest that there is not just a strictly dependence on firing rate and that another firing mechanism is involved.

Interestingly, the other direction selective retinal ganglion cell type, the ON-DSRGC, revealed consistent differences in short ISI distribution and information rates when recorded in response to the same stimulus. This cell type is known to be less direction selective than ON-OFF DSRGC and have larger receptive fields and projects reliably to the accessory optic system (AOS) signaling the global retinal motion [32]. The mechanism used here is different when compared with the mechanism for information transmission used at the retinogeniculate synapse, at least from the point of view of a large convergence of many ON-DSRGCs on a single AOS counterpart cell [33]. Presumably this stands for an explanation concerning the previously mentioned differences between ON DSRGCs and ON-OFF DSRGCs.

In the last set of investigations we checked whether the information regarding visual stimuli carried by individual spikes varies with ISI. We found that the amount of information per spike decreased as the ISI increased. This finding, together with the previously presented results, suggests that ISI-based filtering of retinal spike trains is part of the mechanism that helps in preserving information about the important features of visual stimuli as it travels from retina to cortex, increasing the information efficiency to improve signaling the optimal stimulus features as has been suggested also by recent studies in macaque and cat [22, 23, 29].

## 2. Materials and Methods

### 2.1. White Noise Stimulus

Experiments were performed on whole-mount retinas, in accordance with the animal use committee of the Massachusetts General Hospital. Procedures have been described previously [4, 34, 35].

We analyzed the previously recorded neuronal activity from ON-OFF retinal ganglion cells of the 4 adult isolated rabbit retinas stimulated with white noise and drifting grating bars. A sixty-channel multielectrode array, with a 30 *μ*m spatial resolution (Multichannelsystems, Reutlingen, Germany), was used for electrophysiological recordings. Data acquisition and off-line analysis have been previously described in [4]. Briefly, the receptive field was mapped using white noise stimulus (temporal flat power spectrum in the 1–30 Hz range), which comprised a 16 × 16 array of squares (pixels) with the updating rate of the frames of 50 Hz. The luminance of each square was independently modulated by an m-sequence [36]. The size of each square was 75 *μ*m, and the size of the receptive field of each cell was calculated by reverse correlating stimulus and spike response, considering checkers whose intensity at the temporal maximum of the mean effective stimulus exceeded by a factor of 3 the SD of the squares in the background [37]. The duration of stimulus was *T* = 30 s,  and the stimulus was repeated *n* = 30 times.

### 2.2. Spike-Triggered Analysis

Spike-triggered average (STA) was calculated after the spikes were sorted into different categories according to ISIs values 0 < ISI ≤ 10 ms; 10 < ISI ≤ 20 ms; 20 < ISI ≤ 50 ms; 50 < ISI ≤ 100 ms, and in the last category STA was calculated for all spikes in the spike trains.

We calculated STA as classically defined [38] as the average over all the stimuli which shortly preceded a spike:

(1) $$\begin{array}{c} \text{STA}\left(\tau \right)=\frac{1}{\langle \text{Nsp}\rangle }\langle \sum_{i=1}^{\text{Nsp}}S\left(ti-\tau \right)\rangle , \end{array}$$

where Nsp is the number of spikes, *ti* is the time of occurrence of spike *i*, *s*(*t*) is the stimulus at time *t*, and the angle brackets represent averaging over trials. We represent the spike train *ρ*(*t*) as a sum of infinitesimally narrow, idealized spikes in the form of Dirac *δ* functions:

(2) $$\begin{array}{c} \rho \left(t\right)=\sum_{i=1}^{\text{Nsp}}\delta \left(t-ti\right). \end{array}$$

Thus, STA(*τ*) can be expressed as an integral of the stimulus times the neural response function:

(3) $$\begin{array}{c} \text{STA}\left(\tau \right)=\frac{1}{\langle \text{Nsp}\rangle }\int_{0}^{T}r\left(t\right)s\left(t-\tau \right)dt, \end{array}$$

where *T* is the total duration of a trial and *r*(*t*) is the firing rate at time *t*. The correlation function of the firing rate *r* at time *t* and stimulus *s* at time (*t* + *τ*) is denoted by

(4) $$\begin{array}{c} \text{Corr}{\left(\tau \right)}_{rs}=\frac{1}{T}\int_{0}^{T}r\left(t\right)s\left(t+\tau \right)dt. \end{array}$$

Finally, STA(*τ*) as the correlation between stimulus and neural response was calculated by:

(5) $$\begin{array}{c} \text{STA}\left(\tau \right)=\frac{T}{\langle \text{Nsp}\rangle }\text{Corr}{\left(-\tau \right)}_{rs}=\frac{1}{\langle r\rangle }\text{Corr}{\left(-\tau \right)}_{rs},\\ \text{MCorr}=\max\left(\left|\text{STA}\left(\tau \right)\right|\right). \end{array}$$

Maximum value of STA(*τ*), given by MCorr, was indicating the maximum correlation between stimulus and neuronal response, for each of the ISI categories, and has the dimension of light intensity.

### 2.3. Grating Bars Stimulus

Further on, direction selectivity was tested using a square wave spatial grating moved in 8 equally separated directions with *n* = 7 repetitions for each direction, as previously described [4].

Briefly, the total stimulus length was 672 seconds, consisting in 12 seconds for each direction multiplied by 8 different directions and by 7 different trials. The spatial extent of the moving grating was 2500 *μ*m on the retina and, thus, multiple cells were stimulated and recorded simultaneously.

Individual tuning curves were obtained considering the firing rate of each cell for each of the eight equidistant directions. The firing rates for each cell and for each of the stimulus direction were averaged over the number of stimulus repetitions (seven repetitions of the stimulus presentation were done for each different direction of movement).

In this study we used data from 20 retinal ganglion cells. Three of the cells were ON direction selective (ON DSRGC), twelve of the cells were ON-OFF direction selective (ON-OFF DSRGC), and five of the cells were nondirectional selective (NON-DSRGC).

### 2.4. Direction Selectivity Index

To quantify the directional tuning of a neuron, we used the direction selectivity index (DSi) as described by Taylor and Vaney [30]:

(6) $$\begin{array}{c} \text{DSi}=\frac{||\sum_{i}{\vec{\nu }}_{i}||\;}{\sum_{i}r_{i}},\;{\vec{\nu }}_{i}=r_{i}\left(\begin{array}{c} \cos\varphi_{i}\\ \sin\varphi_{i} \end{array}\right) \end{array}$$

${\vec{\nu }}_{i}$ is a vector pointing in the direction of the stimulus with the length equal to the number of spikes recorded during presentation of the stimulus (*r*_*i*_). The DSi explains the directional tuning based on the firing rates for different particular movement directions of the visual stimulus.

### 2.5. ISI Directional Index

Once we quantified the directional tuning of the recorded ON-OFF RGCs, we asked what influence the ISI distribution may have over the calculated DSi. In this sense we isolated ISIs of each recorded spike trains as response of each direction of stimulus movement again into the following categories: 0 < ISI ≤ 10 ms; 10 < ISI ≤ 20 ms; 20 < ISI ≤ 50 ms; 50 < ISI ≤ 100 ms, and all spikes. To compare the direction selectivity for different ISIs values with the direction selectivity for entire recorded activity we defined the following index as ISI directional index (SI):

(7) $$\begin{array}{c} \text{SI}=\frac{\text{DSi}\left(\text{ISI}\right)}{\text{DSi}\left(\text{DSRGC}\right)}. \end{array}$$

DSi(DSRGC) represents the index of selectivity calculated for all recorded activity as indicated in (5), and DSi(ISI) represents the directional tuning calculated for the separated retinal spikes according with different ISI values, as mentioned previously. This index is quantified similar to the DSi in (5) having into account that for each direction of stimulus movement we took into account the total number of ISIs (of a certain value) instead of total number of spikes.

### 2.6. Burst Distribution

Burst firing events were considered when at least two spikes occurred after an ISI larger than a time threshold of 50 ms and are followed by an ISI shorter than 5 ms [39, 40]. Each burst event was then scanned to calculate the number of spikes per burst. We then formed different burst categories depending on the number of spikes per burst starting with 2 spikes per burst, 3 spikes per burst, and so on up to a number of 10 spikes per burst.

### 2.7. Poisson-Like Spike Trains

If the short ISI activity tuning at preferred direction would be strictly firing rate dependence, one presumes that Poisson-like spike trains with the same firing rate as the recorded spike trains would give a similar ISI distribution. Thus, we generated Poisson-like spike trains with the firing probability equally distributed over time according to a Poisson process with a refractory period of 5 ms [31]. As mentioned before these Poisson-like spike trains hold the same averaged firing rates as the recorded DSRGCs for each of the stimulus direction. Briefly, we computed the Poisson-like spike trains by using Matlab (MathWorks) user-written routines based on the probability that *n* spikes in an interval *T* can be computed according to Poisson distribution, for each of the stimulus direction:

(8) $$\begin{array}{c} P_{T}\left(n\right)=\frac{{\left(rt\right)}^{n}}{n!}\exp\left(-rT\right), \end{array}$$

where *T* = trial duration and *r* = average firing rates for each of the stimulus direction.

The spike times for each of the stimulus direction were then generated by the interspike interval formula and adding the refractory period of 5 ms:

(9) $$\begin{array}{c} t_{i+1}-t_{i}=-\left(\frac{1}{r}\right)\ln\left(\text{Xrand}\right), \end{array}$$

where Xrand is a random number uniformly distributed over the range between 0 to 1 and *t*_*i*_ represents the spike times for *i* = 1, 2,…*n* spikes.

### 2.8. Entropy

To calculate the poststimulus time histograms (PSTHs) we aligned the spike sequences with the onset of stimuli that repeated *n* times. For periodic stimulus (grating bars), we moved the response sequence back to time zero after each time period *T* and counted *n* as the total number of periods of data. We then divided the stimulus period *T* into *N* bins of size Δ and counted the number of spikes *ki* from all *n* sequences that fell in the bin *i*. The optimal bin size Δ results from minimizing (2*k* − *σ*)/Δ^2^, where *k* is the mean of *ki* and *σ* is the variance of *ki* [41].

We then averaged the calculated PSTH for the *n* repetitions of the stimuli (*n* = 30 for white noise stimulus and *n* = 7 for grating drifting bars stimulus) and obtained the time-varying firing rates *r*(*t*).

In order to evaluate the information about the stimulus carried by single spikes we used the previously calculated time-varying firing rates *r*(*t*) and computed the estimates of entropy (*H*) as follows [22, 23, 29, 42, 43]:

(10) $$\begin{array}{c} H=\frac{1}{T}\int_{0}^{T}dt\frac{r\left(t\right)}{\langle r\rangle }\text{lo}{\text{g}}_{2}\frac{r\left(t\right)}{\langle r\rangle }, \end{array}$$

where *T* = 12 s represents each trial duration and 〈*r*〉 represents the average firing rate.

We calculated *H* for *n* = 20 cells for white noise stimulus and *n* = 12 cells for drifting grating bar stimulus and for each of the ISI categories mentioned at the beginning. Additionally for the second stimulus we calculated *H* for each of the eight equidistant directions of stimulus movement. Thus, we could estimate the entropy tuning for each cell and evaluate the amount of information carried at each direction of stimulus movement similarly with (6):

(11) $$\begin{array}{c} \text{DSi}H=\frac{||\sum_{i}^{}{\vec{\nu }}_{i}H||}{\sum_{i}H_{i}},\;{\vec{\nu }}_{i}H=H_{i}\;\left(\begin{array}{c} \cos\varphi_{i}\\ \sin\varphi_{i} \end{array}\right), \end{array}$$

where *H*_*i*_ represents the entropy for each of the stimulus direction of movement.

To take into account the problem of the size limitation of data and to correct the resulting bias, the information rates were estimated by extrapolating correct entropy *H*_*c*_ from segments of the total data, in an increasing order for different bin sizes (∇*τ*) and fit by [42]

(12) $$\begin{array}{c} H\left(T,\nabla \tau \right)=H_{c}\nabla \tau +\frac{H1\left(T,\nabla \tau \right)}{\text{size}}+\frac{H2\left(T,\nabla \tau \right)}{{\text{size}}^{2}}. \end{array}$$

The linear dependence gave a good fit for all cells included in our analysis. This measure of information rates does not make any deduction about the number of relevant stimulus features and let us know about the amount of information (bits/spikes) contained in single spikes.

To check the statistical significant differences among different recorded data (ON-OFF DSRGCs and ON DSRGCs) or generated data (Poisson-like spike trains) we used statistical *t*-test and calculated the corresponding *P* values. In general, the data was summarized over cell types and different trials by using the mean and standard deviation (std).

## 3. Results

### 3.1. Maximum Correlation between Stimulus and Neural Response

**Figure 1:**
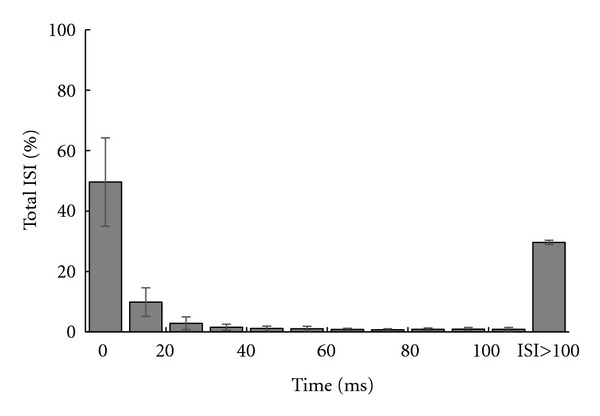
Distribution of mean ISIs for all 20 cells. *Y* axis represents the percentage of all ISIs for each ISI category distributed on *X* axis. Maximum of mean ISI distribution for all cells was at ISI between 0 and 10 ms.

In order to investigate the correlation between visual stimulus and ISI distribution within neural response at the output of retina, we firstly analyzed the previously recorded neural activity from 20 retinal ganglion cells of rabbit retina in response to the white noise visual stimulus. Among them, 5 cells were nondirectional selective (NDS) and 15 of them were directional selective cells (DSRGCs). Scanning the spike trains of each retinal ganglion cell in response to the stimulus applied, we found that for all of the cells most of the spikes were preceded by short time intervals; that is, the activity with short ISI was predominant. Similar results were also previously reported [30].  Figure 1 shows the distribution of mean ISIs for all 20 cells. The majority of the ISIs were below 200 ms. Maximum of mean ISI distribution for all cells was at ISI between 0 and 10 ms. Thus, we found that the largest value of mean ISI is for ISI > 0 and ISI ≤ 10 ms and that it consistently decreases for larger ISI categories (see Table 1, row 1).

**Figure 2:**
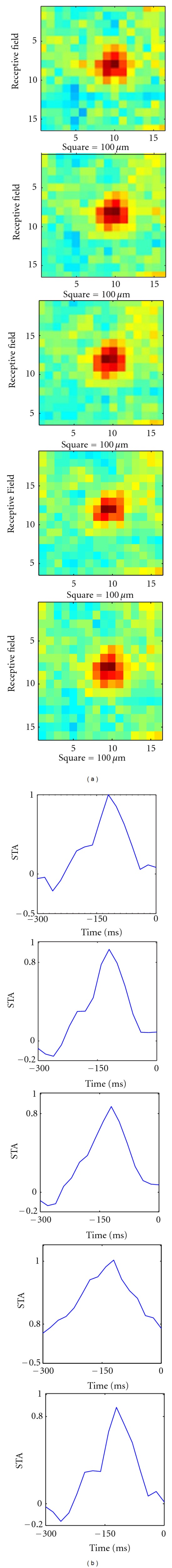
(a) The receptive field of retinal ganglion cells for each of the mentioned ISIs categories. (b) The maximum correlation found between stimulus intensity and recorded spiking activity. First row corresponds to 0 < ISI ≤ 10 ms and presents the maximum correlation between stimulus and neural response. Second row is for 10 < ISI ≤ 20 ms, third row is for 20 < ISI ≤ 50 ms, fourth row is for 50 < ISI ≤ 100 ms, and last row is for all ISIs included. As ISI increases, the maximum correlation between stimulus and neural response decreases.

Recent scientific results suggest that short ISIs in the retinal ganglion cell's spike trains are associated with cell's receptive field shape and stimulus intensity [21]. We used reverse correlation analysis to map the receptive field of each cell for different values of ISI. To do so, we selected all the spikes in each cell's spike train with 0 < ISI ≤ 10 ms, 10 < ISI ≤ 20 ms, 20 < ISI ≤ 50 ms, 50 < ISI ≤ 100 ms and applied reverse correlation analysis to check the correlation between the stimulus intensity and recorded neural response.  Figure 2(a) shows the shape of the receptive field of a recorded DSRGC for each of the above-mentioned ISIs categories, and Figure 2(b) shows the maximum correlation found between stimulus intensity and recorded spiking activity.

Firstly, we noticed that the spatial location of the ISI specific receptive field's center was not changed across the ISIs categories and thus remained fairly the same regardless of the fact that ISIs varied. It is beyond our scope to study in detail how the cells receptive field's size changes as ISI varies. However, it is worth to be noted that we also found slight changes in the size of receptive field of the recorded cells for different ISI categories and for each cell, as previously reported. Instead, our aim is to ask how ISI distribution is correlated with visual stimulus applied. Thus, using reverse correlation we mapped the ISI-specific receptive field and quantified the maximum correlation between stimulus and recorded spiking activity as described by MCorr; see Section 2.2 Methods, (5) (Figure 2(b)).

**Figure 3:**
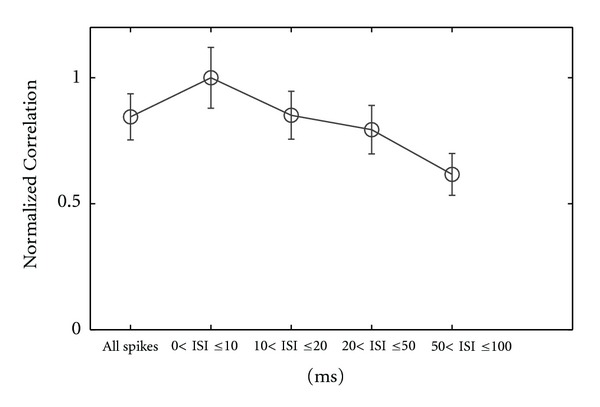
Relationship between normalized correlations (*Y* axis) calculated from the recorded neural response following the stimulus presentation and different ISI categories (*X* axis).

For all cells and cell types we found that maximum correlation between visual stimulus and recorded neural response was for ISIs shorter than 10 ms. As ISIs increase up to 20 ms (i.e., 10 < ISI ≤ 20 ms), the maximum correlation between stimulus and neural response decreases down to about 85% of the value for 0 < ISI ≤ 10 ms and continues to decrease for 20 < ISIs ≤ 50 ms when it becomes slightly lower than maximum correlation found for all spikes in the recorded activity (all ISIs category; see Table 1, row 2).  Figure 3 shows the distribution of normalized mean MCorr for all 20 cells recorded, for each of the ISI category. For this particular investigation we calculated MCorr (5) for each, cell and then we found the mean for all 20 recorded cells and normalized the results. This calculation was repeated for each of the ISI category and for all spikes. Thus, for 0 < ISI < 10 ms normalized mean MCorr has the highest value obtained and continues to decrease as ISI increased (see Table 1, row 2).

It is already known that the efficacy to evoke an action potential (AP) at the postsynaptic target is greatest for spikes preceded by short interspike intervals (ISI ≤ 10 ms). This efficacy decreases as ISI increases so that for ISI longer than 30 ms it has almost no significant influence [3, 18]. This, together with our results, suggests that there is a positive association among maximum correlation between stimulus and ISI of the recorded spike trains and the efficacy of evoking an AP at the postsynaptic target. In this logic ISI-based filtering of retinal spike trains varies with stimuli and helps preserving particular visual information, which might be of a maximum significance, toward the next stage within early visual system. We next checked if this association is robust for different visual stimulus. To do so we analyzed the recorded neural activity of the retinal ganglion cells in response to drifting grating bars, moving in 8 different directions. This stimulus was chosen due to the fact that for direction selective retinal ganglion cells drifting grating bars moving in the preferred directions represent the optimal stimulus feature and thus evoke the maximum response. If this ISI-based filtering holds true, the information regarding the preferred direction of stimulus movement should be preserved against the intermediate directions.

### 3.2. Direction Selectivity and ISI-Based Filtering

**Figure 4:**
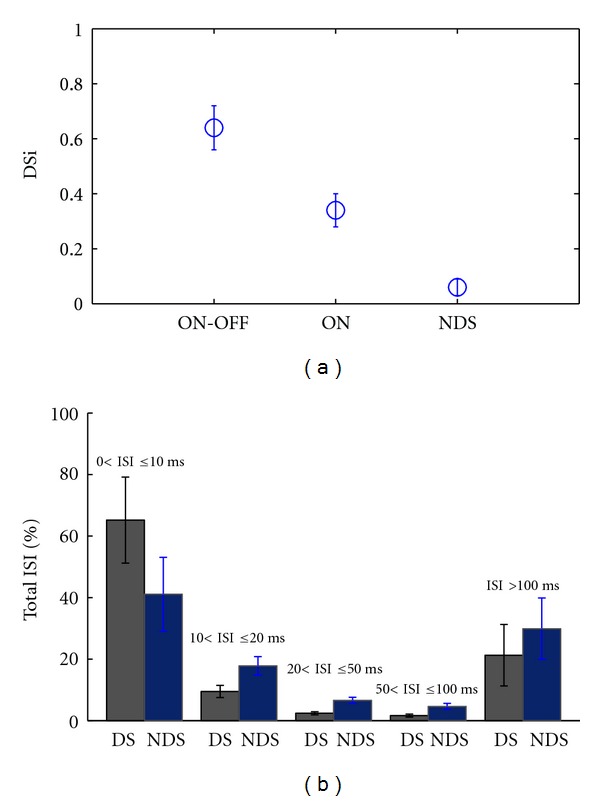
(a) Index of Selectivity (DSi) for three different types of the recorded cells in response to moving grating bars. (b) Total ISI distribution for directional selective neurons (DS) gray bars and nondirectional selective neurons (NDS) blue bars.

In the next step we analyzed the recorded neural activity in response to a different stimulus consisting in drifting grating bars (see Section 2.3 Methods). At each direction of stimulus motion we recorded the cells' responses consisting in different spike trains. Thus we calculated the directional tuning of each cell and quantified it as directional selectivity index (DSi), as described in Methods Section 2.2, (6). Twelve of the cells were ON-OFF directional selective (ON-OFF DSRGCs), and three of the cells were ON direction selective (ON DSRGC), while the other five cells were nondirectional selective (NDS). The mean index of selectivity for ON-FF DSRGCs was DSi = 0.64 (std = 0.08, number of cells = 12) and for ON DSRGC the mean DSi = 0.34 (std = 0.06, number of cells = 3) while for NDS we found that mean index of selectivity was DSi = 0.06 (std = 0.01, number of cells = 5), (Figure 4(a)) in accordance with previously reported results [4, 30, 31].

The results, using white noise stimulus and reverse correlation, indicated that shortest ISI spiking activity was associated with maximum correlation between stimulus and recorded spiking activity in response to the stimulus presented. That is, when short ISIs are present one would expect that the stimulus applied influenced at maximum the receptive field of the cell and thus the probability that the cell transmits further on that particular information about the stimulus. Direction selective cells have the property to respond vigorously at the preferred direction of stimulus movement and are silent for the opposite null direction. Thus, the stimulus feature of maximum importance for the ON-OFF DSRGCs is the direction of stimulus motion.

We firstly scanned all the spike trains of each cell, quantified the ISI distribution, and found that for ON-OFF DSRGCs the peak is obtained for 0 < ISI < 10 ms and is significantly higher than for the peak obtained for the rest of the RGCs (ON-DS and NDS) which did not show a clear prominent peak in ISI distribution. The short ISI spiking activity for all ON-OFF DSRGCs shows a mean ISI of 65.2% for 0 < ISI ≤ 10 ms, decreasing as ISI increases (see Table 1, row 3). By contrary, NDS presented a lower mean ISI value for 0 < ISI ≤ 10 ms than for directional selective cells, about 41.1% of total ISIs. As ISI increases, we noticed higher mean ISI for NDS as compared with ON-OFF DSRGCs (see Table 1, row 4 and Figure 4(b)). ISI spiking activity is more concentrated on short values for directional selective cells than for NDS where ISI spread out toward higher values.

Further on, we scanned again the recorded spike trains of all cells and selected into separate categories, spiking activities containing 0 < ISI ≤ 10 ms, 10 < ISI ≤ 20 ms, 20 < ISI ≤ 50 ms, and 50 < ISI ≤ 100 ms. For each of these selected spike trains we calculated the directional tuning and quantified it again with an index of selectivity similar as in (6) (see Section 2.4 Methods).

Firstly, we noticed that for ON-OFF DSRGCs the highest activity with short ISI was distributed at preferred direction and thus correlating the short ISI spiking activity with signaling the direction of stimulus motion.

We found the highest DSi for the spiking activity of the ON-OFF DSRGCs with 0 < ISI ≤ 10 ms. Moreover, once that ISI, increased the DSi decreased. That is, the shorter the ISI is, the better direction of stimulus movement is signaled by the ON-OFF DSRGCs.

**Figure 5:**
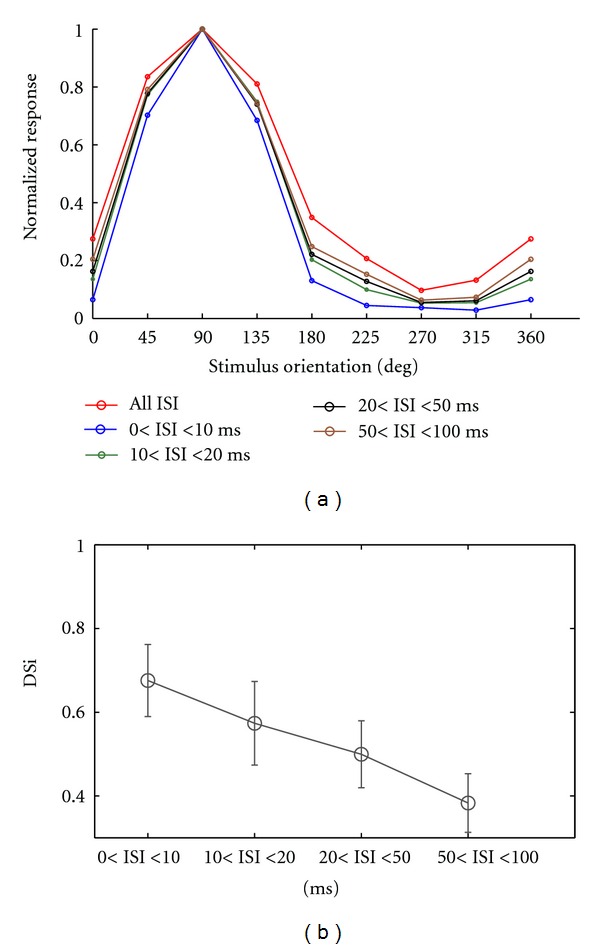
(a) Example of sharpening in tuning curves for a recorded ON-OFF DSRGC for different ISI categories. (b) Distribution of DSi for all recorded ON-OFF DSRGCs for all ISI categories.

Indeed, Figure 5(a) shows how sharpening in direction selectivity is produced for spikes with short ISIs. Thus for the ON-OFF DSRGC exemplified in Figure 5(a), for 0 < ISI ≤ 10 ms, we found DSi = 0.70 and decreases as ISI value increases, such as for 10 < ISI ≤ 20 ms, DSi = 0.62, for 20 < ISI ≤ 50 ms DSi = 0.59, for 50 < ISI ≤ 100  DSi = 0.56 and for all spikes DSi = 0.49.

The decreasing in index of selectivity as ISI increases was found for all twelve recorded ON-OFF DSRGCs (Figure 5(b), Table 1, row 5).

**Figure 6:**
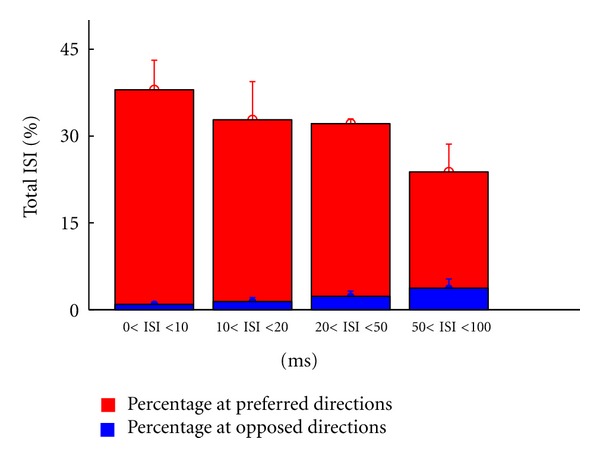
Total ISI distribution for all recorded ON-OFF DSRGCs, at preferred, and opposite directions of stimulus movement, for all ISI categories.

Furthermore, Figure 6 depicts the ISI distribution for all ON-OFF DSRGCs reflecting the finding that short ISI activity is focused at preferred directions of stimulus motion. We found that for 0 < ISI ≤ 10 ms, the largest percentage from all ISIs of this category was at preferred direction. At opposite direction of stimulus motion we found only a small percentage of all ISIs in this category.

In the next ISI category, 10 < ISI ≤ 20 ms, from the total of ISIs that belong to this category we found at preferred direction also a larger percentage than at opposite direction. However, one can observe a slight increasing in 10 < ISI ≤ 20 ms spiking activity at opposite direction. This trend is robust up to the last ISI category (see Table 1, row 6).

Thus we found that the percentage of short ISIs at preferred direction becomes lower as ISI category increases and the percentage of short ISIs at opposed direction increases as ISI category increases. In this way the decreasing of DSi noticed previously (Figure 5(b)) as ISI increases might have an explanation in this distribution of short ISI activity at preferred direction. As ISI increases, we found increasing short ISI activity at the intermediate and opposed directions too, and thus the selectivity becomes weaker (Figure 6).

**Figure 7:**
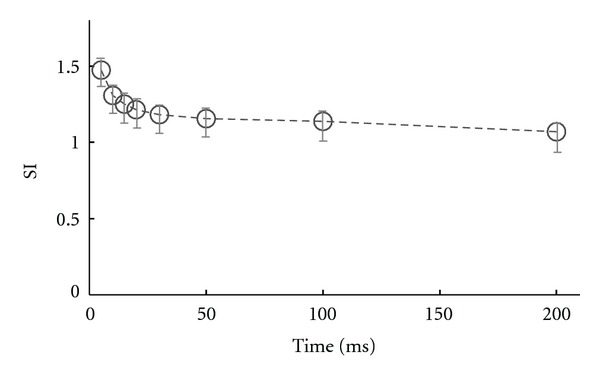
ISI Directional Index (SI) variation for all ON-OFF DSRGCs.

In the next set of investigations, to better exemplify the relation between ISI and directional tuning, we quantified the ISI directional index (SI, (7) Section 2.5 Methods) as a measure of directional selectivity for each ISI category in comparison to the entire spike train.  Figure 7 shows how this index varies with different ISI values. Briefly, for ON-OFF DSRGCs the best sharpening is observed at ISI = 5 ms where SI = 1.47 (std = 0.09, number of cells = 12) and decreases as ISI increases, down to 1, which means that signaling the direction of stimulus motion is as good as for all spikes in the spike train.  SI = 1.30 (std = 0.08, number of cells = 12) for ISI = 10 ms, SI = 1.25 (std = 0.08, number of cells =12) for ISI = 15 ms.

### 3.3. Firing Rate Dependence

So far, we have seen by applying white noise and reverse correlation analysis that for different ISI values the correlation between stimulus and neural response varies and that a maximum correlation between stimulus and neural response is obtained for the shortest ISI category (0 < ISI ≤ 10 ms). We then noticed that for a different stimulus, drifting grating bars, short ISI spiking activity was focused at preferred direction (optimal stimulus feature for DSRGCs) and that index of directional selectivity was decreasing as ISI was increasing. Our results, together with the already known paired spike efficacy and the major influence of short ISI activity in signaling information about visual stimulus at different synapses along early visual system [18, 22, 23, 29], bring us to the hypothesis that ISI temporal filtering might be part of a mechanism responsible for preserving information in the transmission process from retina to LGN.

Further on, to check whether it is just a strict dependence of short ISI activity at optimal stimulus feature by the firing rate or presumably another mechanism is involved (i.e., burst firing), we constructed Poisson-like spike trains with similar firing rate and tuning as the recorded ON-OFF DSRGCs (see Section 2.7 Methods) [31].

**Figure 8:**
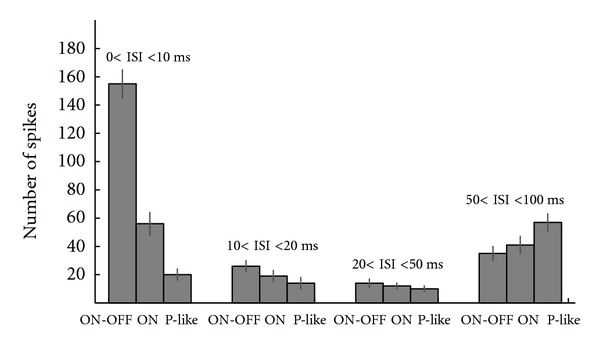
Distribution of spikes for all ISI categories, in number of spikes at preferred directions, for ON-OFF, ON, and Poisson-like spike trains.

**Figure 9:**
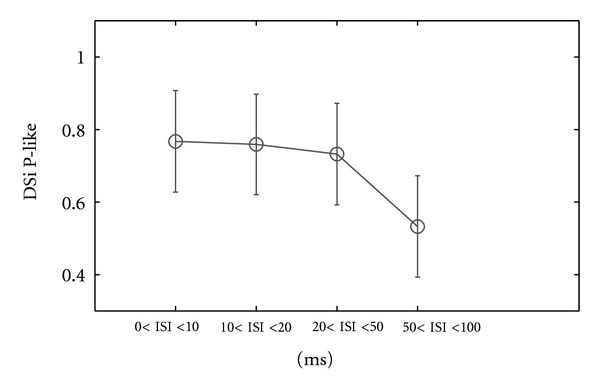
Variation of DSi for Poisson-like spike trains versus different ISI categories.

Figure 8 shows the number of spikes for each of the ISI categories at preferred direction of stimulus movement. For ON-OFF DSRGCs we noticed at preferred direction within 0 < ISI ≤ 10 ms category the largest mean number of spikes, while for Poisson-like spike trains with similar firing rates and DSi we found a lower mean number of spikes at the preferred directions (see Table 1, rows 8, 9, and 10). Furthermore, for all ISI categories we noticed that the mean number of spikes was different comparing ON-OFF DSRGCs with simulated P-like spike trains. Since the Poisson-like spike trains have the same firing rates and tunings as the recorded ON-OFF cells, one would expect similar number of spikes for each ISI category. By contrary we found a statistically significant difference (*P* < 0.02) between the short ISI distribution of the ON-OFF recorded spike trains and the Poisson-like spike trains. The difference that our findings show consistently indicates that the increase in number of spikes for short ISI spiking activity cannot be predicted by stochastic Poisson process and that another mechanism should be involved within distribution of short ISI spiking activity, and thus it is not just a strict firing rate dependence. Additionally, for Poisson-like spike trains the short ISI spiking distribution did not show large differences between different ISI categories and was significantly larger just for ISI > 50 ms. This could also explain the direction selectivity which has also an almost uniform distribution for different ISI categories except for the largest one (Figure 9 and Table 1, row 11).

The results from the Poisson-like spike trains indicate the degree of directional tuning does depend on the short ISI activity at preferred direction encountered in ON-OFF direction selective cells. It does not depend on the neuron's firing rates that are different for the different recorded DSRGCs and Poisson-like spike trains (compare Figure 5(b) and Figure 9).

### 3.4. Information Rates

In the next set of investigations we checked how much information regarding visual stimuli each different ISI category carries on. Not only for white noise stimulus but also for drifting grating bars shortest ISI category, 0 < ISI ≤ 10 ms, carried the highest information rate. As ISI increased, we found lower amount of information within the each increasing ISI category.

**Figure 10:**
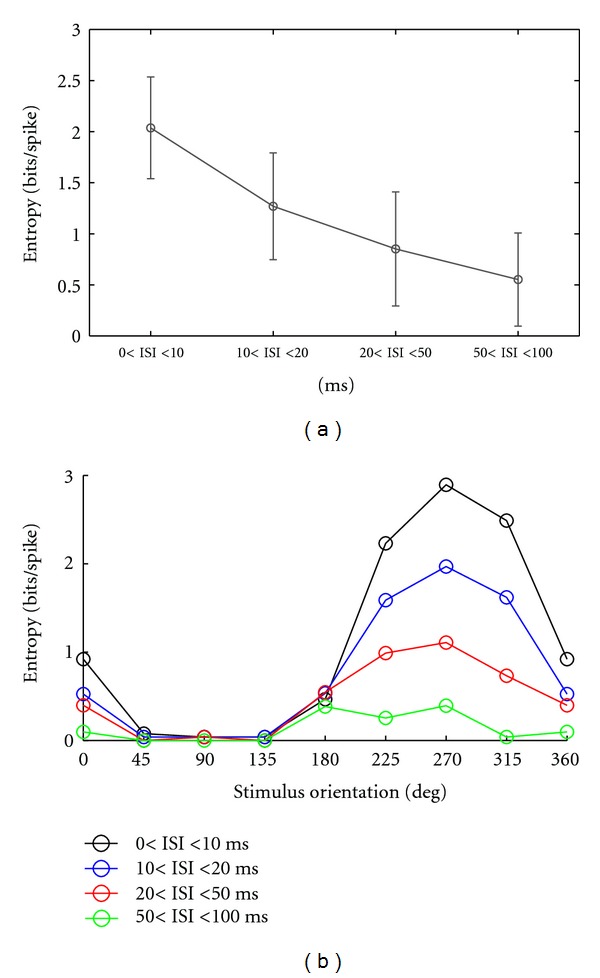
(a) Variation of entropy in bits/spikes for all ON-OFF DSRGCs versus different ISI categories. (b) An example of entropy (bits/spike) tuning curve for one recorded ON-OFF DSRGC for all ISI categories.

We calculated the entropy for all 12 recorded ON-OFF DSRGCs and for each ISI category, firstly for the drifting grating bars stimulus (Figure 10(a)). We found that maximum entropy was achieved for shortest ISI category (see Table 2, row 1) as ISI increased the amount of information about visual stimulus decreased.

Using (11) (see Section 2.8 Methods), similarly to index of directional selectivity for firing rates, we calculated the tuning of entropy for all ISI categories.  Figure 10(b) shows one example of an ON-OFF DSRGC and indicates that as ISI decreases, the information tuning becomes more sharpened.

**Figure 11:**
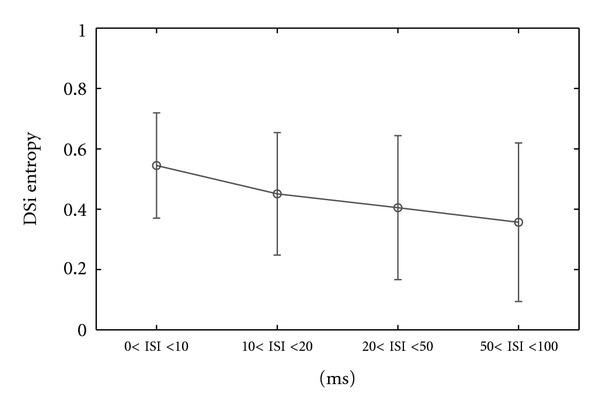
Variation of index of selectivity (DSi) of entropy for all recorded ON-OFF DSRGCs and for all ISI categories.

For all 12 recorded ON-OFF DSRGCs we found that index of selectivity for entropy (DSiH) decreased as ISI increased (see Figure 11 and Table 2, row 2).

Altogether, we found for all twelve ON-OFF DSRGCs that the amount of information regarding visual stimulus was highest for shortest ISI category and was tuned at preferred direction of stimulus movement.

**Figure 12:**
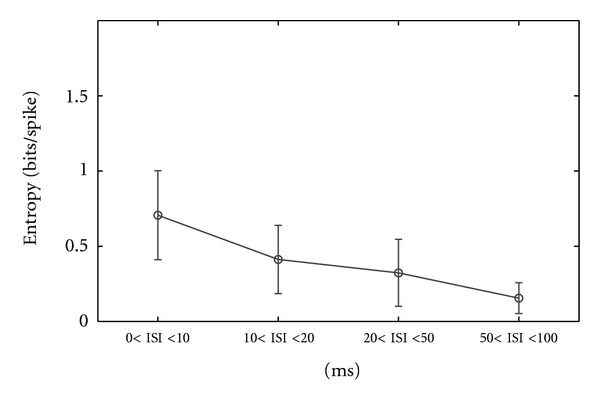
Variation of entropy (bits/spike) for all 20 RGCs recorded using white noise stimulus versus different ISI categories.

Finally, for white noise stimulus we noticed the same trend in decreasing the information rates as ISI increased (Figure 12 and Table 2, row 3).

Consistent with our previously mentioned results, these findings suggest that the ISI-based filtering of retinal spikes is part of the mechanism of information processing that recodes the visual signal using a sparse coding [44], to improve the overall coding efficiency from one stage to another within the visual system.

### 3.5. Comparison between ON-OFF DSRGCs and ON DSRGCs

#### 3.5.1. ISI Distribution

As we have already seen in Figure 8, the two types of DSRGCs, the ON and ON-OFF, show statistically significant differences (*P* < 0.01) concerning the ISI distribution within their recorded spike trains. ON DSRGCs responded to the stimulus presentation at preferred direction with only around one-third of the number of spikes as compared with ON-OFF DSRGCs for the shortest ISI category. For the intermediate ISI categories (10 < ISI < 20 and 20 < ISI < 50 (ms)) ON and ON-OFF DSRGCs showed a similar number of spikes in each ISI category. Moreover for the largest ISI category, we found for the ON DSRGC a slightly larger number of spikes than for ON-OFF DSRGC.

**Figure 13:**
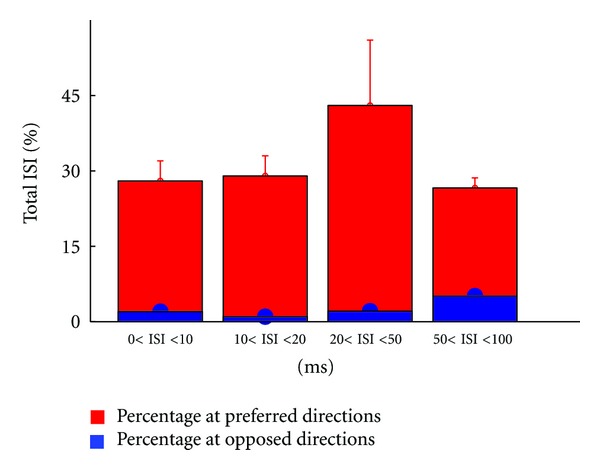
Total ISI distribution for all recorded ON DSRGCs, at preferred, and opposite directions of stimulus movement, for all ISI categories.

**Figure 14:**
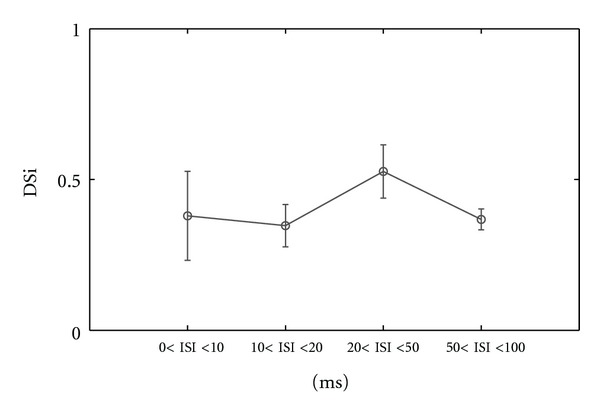
Distribution of DSi for all recorded ON DSRGCs for all ISI categories.

Comparing Figure 6 with Figure 13 we notice the differences of ISI distribution for each ISI category between ON-OFF and ON cell types. Thus, for ON cells we found at preferred direction (Figure 13 and Table 2, row 4) for 0 < ISI ≤ 10 ms a percentage of 28.12% (std = 4.2, number of cells = 3) from all ISIs of this category which represents 10% lower number of ISI than ON-OFF DSRGCs (Figure 6). At opposed direction we found a percentage of 2.1% (std = 0.4, number of cells = 3) from all ISIs of this category, for ON DSRGCs which is larger than for ON-OFF DSRGCs (see Table 2, row 5). Thus, within shortest ISI category, ON DSRGCs have lower activity at preferred direction than ON-OFF DSRGCs and higher activity at opposed direction, a discrepancy which may explain the weaker direction selectivity for ON DSRGCs than for ON-OFF DSRGCs. Interestingly for 20 < ISI < 50 ms category we found at preferred direction the largest percentage of total ISIs (Figure 13 and Table 2, row 4) which suggests that ON DSRGCs preferentially use this intermediate ISI category instead of shortest ISI category at preferred direction to signal the stimulus motion direction. By contrary for ON-OFF DSRGCs the shortest ISI category was found as having the highest percentage at preferred direction (Figure 6). This difference is enhanced further on when calculating the index of selectivity for each of the ISI category (Figure 14 and Table 2, row 6). The highest direction selectivity was found for 20 < ISI < 50 ms category (DSi = 0.52, std = 0.08, number of cells = 3) since for the rest of the ISI categories the directional selectivity remains almost constant around 0.3. For ON-OFF DSRGCs a different situation was encountered (Figure 5(b)). The highest direction selectivity was found for shortest ISI category and decreased as ISI category increased.

#### 3.5.2. Burst Distribution

**Figure 15:**
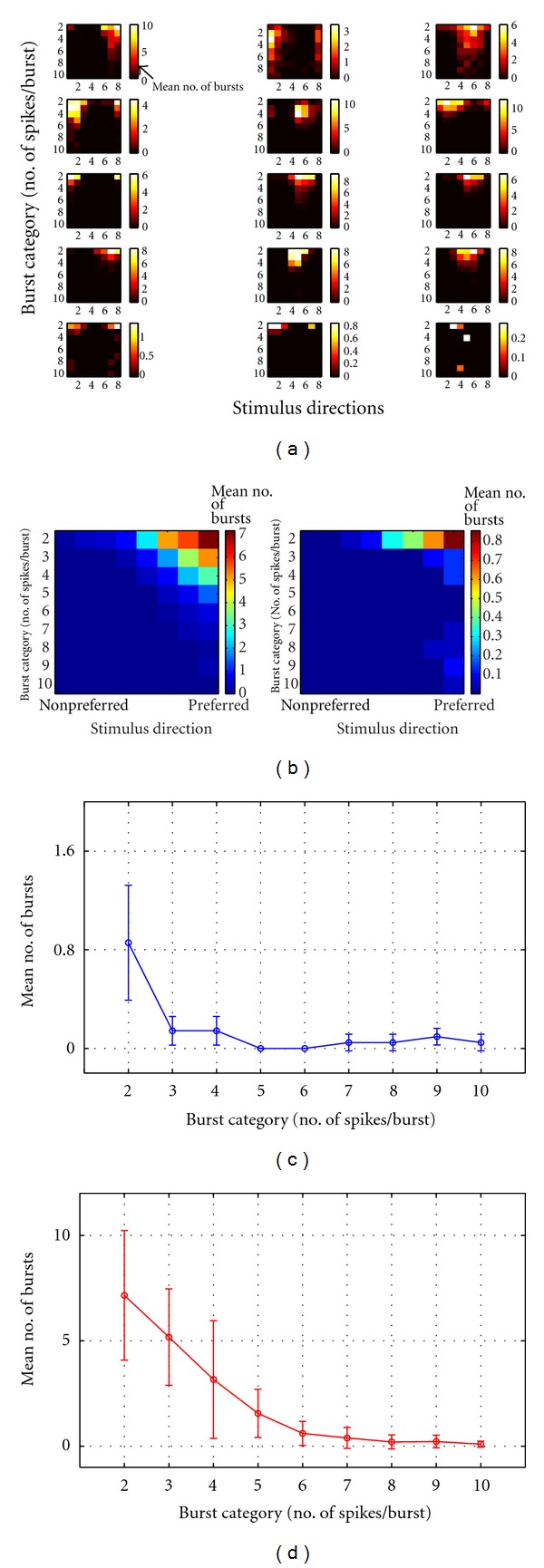
(a) Color coded burst activity for each of the recorded cells. First four rows represent each of the 12 recorded ON-OFF DSRGCs since the last row stands for the three recorded ON DSRGCs. The color code represents the mean number of bursts per trial. For each square the *Y* axis depicts the burst category starting with first row as 2 spikes/burst followed by increasing number of spikes per burst up to the last category consisting in bursts with 10 spikes per burst. *X* axis for each square represents the eight different stimulus directions of movement. (b) Left color map represents the mean burst activity for all 12 ON-OFF DSRGCs. *Y* axis represents the different burst categories in number of spikes/burst. *X* axis represents the stimulus directions. Right color map represents the same for all 3 ON DSRGCs. (c) Mean number of bursts for each of the burst category (in number of spikes/burst) at preferred direction for all ON DSRGCs. (d) Mean number of bursts for each of the burst category (in number of spikes/burst) at preferred direction for all ON-OFF DSRGCs.

The differences between the two cell types regarding the burst activity consisted not only in the mean number of burst at preferred direction but also in mean number of spikes per burst.  Figure 15(a) shows the colored coded distribution of mean number of spikes per burst for each cell. In the first four rows of squares are the ON-OFF cells and in the last row are the three ON cells. For each square the *X* axis depicts the stimulus directions and the *Y* axis represents the burst category as the number of spikes per burst. Thus, first row in each square represents the mean number of bursts with two spikes per burst for each of the eight different stimulus direction. Second row represents the mean number of burst with three spikes per burst for each direction and so on to the last row which represents the mean number of bursts with 10 spikes per burst for each direction.

Shortest burst category, consisting in 2 spikes per burst, was preferred by both cell types as spiking activity in response to stimulus presentation. However, for ON-OFF cells the mean number of bursts within this burst category (with 2 spikes per burst) was larger than for ON DSRRGCs. Interestingly, ON DSRGCs did not respond to the stimulus presentation with bursting activity consisting in more than 2 spikes per burst, unless occasionally. By contrary ON-OFF DSRGCs showed consistent burst-like activity with bursts having more than 2 spikes per burst. Thus, for each ON-OFF DSRGCs we found bursts consisting in 3 spikes per burst up to 6 spikes per burst concentrated at the preferred directions. Indeed, burst categories with 8, 9, or 10 spikes per burst were rarely used not only by ON DSRGCs but also by ON-OFF DSRGCs (Figure 15(a), last rows of each square). The mean number of bursts for each burst category is shown in Figure 15(b) as the mean for all 12 ON-OFF DSRGCs, left side image, and the mean for all 3 ON DSRGCs, right side image. One can notice how ON-OFF DSRGCs used not only short bursts (with 2 spikes per burst) but also larger bursts (with 2, 3, 4, 5, and 6 spikes per burst) in order to signal the direction of stimulus motion. By contrary, ON DSRGCs responded mostly with shortest burst category, consisting in 2 spikes per burst. Additionally for ON-OFF DSRGCs we found large bursts (bursts with more than 2 spikes) predominantly at preferred direction since at opposed and intermediate directions we found a lower number of bursts mostly consisting in 2 spikes per burst.

Figures 15(c) and 15(d) shows how ON DSRGCs use predominantly shortest burst category, with 2 spikes per burst at preferred direction. The mean number of bursts consistently decreased for bursts with more than 2 spikes; mean number of bursts with 2 spikes represented a percentage of almost 62% of total bursts at preferred direction since for bursts with 3 and 4 spikes per bursts we found a very low percentage of around 10% of total bursts at preferred direction. For larger bursts we found 0% for 5 and 6 spikes per burst and only around 3% for bursts with more than 6 spikes.

For ON-OFF DSRGCs the largest percentage was 38%, also for the shortest burst category, but did not decrease abruptly for larger burst where we found 27% for 3 spikes per burst and 18% percent for 4 spikes per burst and 8% for 5 spikes per burst. Larger bursts were rarely accounted and summed remained below 10% of total bursts at preferred direction.

### 3.6. Information Rates

**Figure 16:**
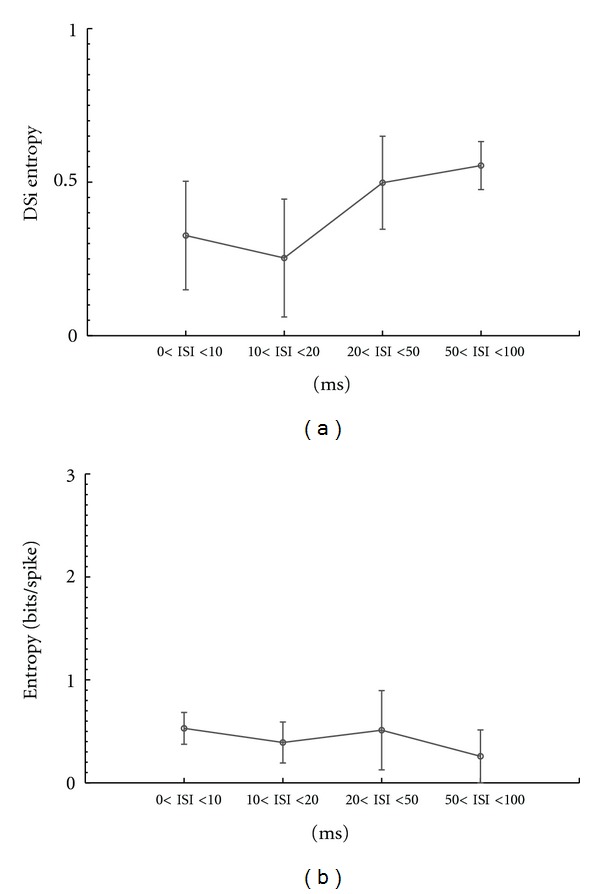
(a) Variation of index of selectivity (DSi) of entropy for all recorded ON DSRGCs and for all ISI categories. (b) Variation of entropy (bits/spike) for all ON DS RGCs recorded using grating bar stimulus versus different ISI categories.

The differences between ON-OFF DSRGCs and ON DSRGCs were also statistically significant (*P* < 0.02) regarding the information about stimulus contained by each ISI category. Figure 10(a) shows that as ISI increased, we found for ON-OFF DSRGCs lower amount of information within each increasing ISI category. For ON DSRGCs Figure 16(b) and Table 2, row 8 show that for two of the ISI categories, namely, 0 < ISI < 10 ms and 20 < ISI < 50 ms, the entropy was almost the same, around the value *H* = 0.52.

This suggests not only that the entropy did not decrease as ISIs increase (as it happened for ON-OFF DSRGCs) but also that the highest amount of information for ON DSRGCs was comparable with the lowest amount of information found for ON-OFF DSRGCs at largest ISI category, *H* = 0.55 (std = 0.4, number of cells = 12).

Additionally for ON DSRGCs, the index of selectivity for entropy DSiH (Figure 16(a) and Table 2, row 7) was highest for the largest ISI category 50 < ISI < 100 ms and not for the shortest ISI category as it was noticed for ON-OFF DSRGCs.

## 4. Discussion

Retinal ganglion cells represent the output of the retina toward higher brain areas, encoding in their spike trains the representation of the visual stimuli which act upon their receptive fields. It is already well known that their firing rate is an important parameter to consider how the relation between stimulus and RGC response has to be characterized. However, many scientific evidence suggests that spike timing, within RGCs spike trains, is another parameter which influences how information is transmitted from retina to the lateral geniculate nucleus (LGN), which represents the next stage in early visual stimulus [22, 23, 29]. Most of the neurons postsynaptic to an RGC, in the LGN, fire about half the number of incoming number of retinal counterparts' spikes, in the process of editing the input spike trains [25, 29, 45, 46].

Moreover, it has been showed that the precise time between two spikes, within an RGC's spike train, is crucial in defining the success of triggering an AP at the postsynaptic target in the LGN. Scientific studies demonstrated that within retinal spike trains, spikes following an ISI lower than 30 ms are more effective than spikes following longer ISIs in evoking an AP at their LGN counterparts [18, 24]. Within this time scale, the temporal summation of excitatory postsynaptic potentials (EPSPs) is mediated mostly by NMDA current so that almost all EPSPs add together to bring the membrane potential of the postsynaptic cell to the spike threshold, with a stronger efficacy at more depolarized membrane potentials [47].

By contrary, the retinal spikes with ISI larger than 30 ms induce a source of noise into the retinal filter that lowers their information capacity [23]. In this sense, the constraint imposed by this temporal summation of closed in time EPSPs selects the stimulus features to those that can evoke such EPSPs sequence and thus bring the LGN cell to the spike threshold. Thus, the transmission of visual stimulus toward the cortex is refined, irrelevant stimulus features are excluded, and consequently LGN cells preserve the important information firing less number of spikes.

This ISI-based filtering presumably represents part of the robust mechanism to process visual information from retina to higher brain areas [21] and lets us know more information about visual stimulus with less number of spikes at postsynaptic counterpart within retinogeniculate synapse [23, 29].

Having these into account we asked how this ISI-based temporal filter is related to signaling new stimulus or important features of the visual stimulus which acts upon the RGCs' receptive fields.

Across our retinal ganglion cells sample, firstly, we found that their spike trains were consistently arranged in periods with high firing rate and interposed periods with isolated spikes [4, 48]. Most of the ISIs were shorter than 200 ms, and the peaks in the ISI histograms were found for ISI shorter than 30 ms for both types of visual stimulus we used.

The most important result using white noise stimulus was that we found the maximum correlation between stimulus and RGCs neural activity for shortest ISI category (ISI ≤ 10 ms). As ISI increased, the maximum correlation between stimulus and cells' response decreased so that for ISI > 30 ms the correlation dropped below the value found for all ISIs in the spike trains.

Short ISI spiking activity (ISI < 10 ms) apparently represents the cells response to the optimal feature of the visual stimulus presented, and presumably the LGN cell counterpart is about to use this ISI-based filtering in order to refine the visual information within its information processing toward higher brain areas [23].

Retinogeniculate synapses have the great advantage of the one-to-one connection between retinal cells and their LGN cells counterpart (an LGN cell has a single retinal main driver acting upon the center of the receptive field and only up to five retinal afferents which affect the surroundings and have a weak influence), and thus the spike timing within the spike trains is of a major importance in information processing. At higher synapses, that is, LGN to V1, the convergence of many more cells acts together to bring the V1 cell to spike threshold [17–19].

To further investigate the influence of the ISI on signaling the optimal stimulus feature, we analyzed the recorded neural activity in response to a different stimulus consisting in drifting grating bars moving in different equidistant directions. This type of stimulus has been extensively used as being a relevant stimulus for quantifying the direction selectivity of DSRGCs [4, 7, 30] having as optimal stimulus feature the direction of stimulus movement. We found that short ISI activity was higher for ON-OFF DSRGCs than for the other RGC types. Additionally, the short ISI activity was tuned at preferred direction of stimulus movement for all recorded ON-OFF DSRGCs.

Another interesting finding was that the direction selectivity index for all ON-OFF DSRGCs was the best for shortest ISI category and decreases as ISI increases. This result is strengthening the idea that directional information is better signaled for shortest ISI. An explanation for this DSi distribution was given by the ISI distribution for each of the ISI categories. For the 0 < ISI ≤ 10 ms most of the short ISIs were focused at preferred direction and thus improving the DSi. As ISI increased, the distribution of short ISI activity was less focused at preferred direction, and thus the difference between preferred, intermediate, and nonpreferred direction diminished resulting in lower DSi.

That ISI-based filtering influencing the signaling of directional information is supported also by the finding that ISI Directional Index (a measure of directional selectivity of each ISI category in respect to all spikes recorded in a spike train) consistently decreased as ISI increased.

Short ISI distribution cannot be predicted by simply increasing firing rate in a stochastic manner as shown by discrepancy between recorded ON-OFF directional selective cells and the Poisson-like spike trains which mimic the recorded cells. Previous scientific results have also shown that short ISI spiking activity is not strictly dependent on firing rates and that firing events of retinal ganglion cells have a higher precision than firing statistics expected by a purely Poisson spike generator [22, 49].

 Presumably for ON-OFF DSRGCs beyond the firing rate, burst-like firing activity plays a key role in explaining how these neurons encode the visual world in discrete firing events [31, 50]. The impact of burst-like activity was previously demonstrated also at thalamocortical synapse [51]. Burst-like firing together with temporal summation and spike threshold acts as mechanism to sharpen not only direction selectivity [31] but also the selectivity for other stimulus features (i.e., orientation selectivity in V1) at different stages within the early visual system [19, 52], at retinogeniculate synapse, [3, 18, 20], at geniculocortical synapse, [53–55]; orientation selectivity—[53, 56]; direction selectivity—[53–56].

Interestingly, for the other direction selective RGC type, the ON direction selective cell, we found less short ISI spiking activity at preferred direction. For this cell type we also found a lower index of selectivity too. Additionally, ON DSRGCs show the highest spiking activity for larger ISI category and also lower information rates than the ON-OFF DSRGCs. The differences between the ON-DSRGCs and the ON-OFF DSRGCs have been strengthened by the dissimilarity noticed in bursts length, since for ON-OFF DSRGCs we found consistent bursting activity with burst categories up to 6 spikes per burst, and for ON DSRGCs we mostly noticed short bursts with two spikes per burst. To account for an eventual improvement in directional information transmission at the output of ON DSRGC (which projects to the Accessory Optic System) presumably the polysynaptic convergent connectivity arrangement must be taken into account [32, 33, 57].

Finally, our last results show that the amount of information regarding the visual stimulus was highest at shortest ISI category and decreased as ISI increased. Moreover the entropy was tuned at preferred direction of stimulus motion having an index of selectivity which decreased as ISI increased. These findings clearly show that most of the information regarding visual stimulus is carried by shortest ISI and is robustly correlated with the preferred stimulus feature.

Our results are in the same trend with other results from recent studies which have shown that the amount of information carried by the LGN cell spike train could be even similar to that of its retinal counterpart but with about half number of spikes for the relay spike train [23, 29]. These findings suggest that at the output of LGN cell the retinal information is represented in a sparse form and thus with an increasing efficiency. Another recent scientific evidence supports this idea and shows that the average information conveyed by a single spike increases across the retinogeniculate synapse by selectively transmitting retinal spikes with the most information [22].

It is already well known that sparse coding used by neurons increasingly from one stage to another is of a fundamental importance concerning coding efficiency, energy efficiency, speed of information, and processing and increasing the storage capacity of memory [44].

As opposed to the retinogeniculate synapse which holds the major advantage of one-to-one connection between retinal ganglion cell and its LGN cell counterpart and thus makes it easier to study the role of ISI-based filtering in process of information transmission, at higher stages, that is, V1, the large polysynaptic connectivity mechanism set hurdles in evaluating the ISI influence on the visual information processing. However, some scientific results support the idea that ISI-based filtering plays a role in information transmission in visual cortex and that they are also consistent with other types of decoding schemes that do not make use of ISIs (averaging the firing rates across many neurons that convey similar information).

## 5. Conclusions

Analyzing the previously recorded neural activity of different types of retinal ganglion cells, we learned out how ISI-based filtering of RGCs spike trains helps in preserving the information regarding the optimal stimulus feature. Maximum correlation between stimulus and neural response is at shortest ISI spiking activity [21].

Short ISIs carry the most information, are focused at optimal (preferred) stimulus feature (in our case direction of stimulus motion), and are not strictly related to firing rate. ISI filtering of spiking activity helps in preserving information related to optimal stimulus feature in transmission from one stage to another within hierarchical brain areas. At presynaptic level already, ISI-based filtering is a part of a mechanism that sharpens the information from one stage to another along the early visual system. This mechanism is prominent for ON-OFF DSRGCs which form one-to-one connections with their postsynaptic target. However it is less evident for ON-DSRGCs which form massive convergence of synaptic inputs upon their postsynaptic target in AOS.

## Figures and Tables

**Table 1:** Summary of ISI distributions and number of spikes for all cells.

	0 < ISI < 10 (ms)	10 < ISI < 20 (ms)	20 < ISI < 50 (ms)	50 < ISI< 100 (ms)	No. of cells
(1)Mean ISI	49.6	9.89	4.1	3.6	20
* *(std) (percentage of total ISI %)	(14.6)	(4.73)	(1.8)	(1.2)
(2)Mean MCorr	0.85	0.79	0.61	0.84	20
* *(std)	(0.09)	(0.09)	(0.08)	(0.09)
(3)Mean ISI	65.2	9.41	2.41	1.65	12
* *(std) ON-OFF DSRGCs (percentage of total ISI %)	(15.1)	(4.2)	(0.8)	(0.5)
(4) Mean ISI	41.1	17.8	6.6	4.62	5
* *(std) NDS (percentage of total ISI %)	(12.8)	(7.1)	(2.1)	(0.8)
(5)Mean DSi ON-OFF DSRGCs	0.67	0.57	0.49	0.38	12
* *(std)	(0.08)	(0.1)	(0.08)	(0.07)
(6)Mean ISI at preferred directions	38	32.8	32.16	23.8	12
* *(std) ON-OFF DSRGCs (percentage of total ISI %)	(5.1)	(6.6)	(0.8)	(4.8)
(7)Mean ISI at opposed directions	0.9	1.4	2.3	3.7	12
* *(std) ON-OFF DSRGCs (percentage of total ISI %)	(0.4)	(0.7)	(0.9)	(1.6)
(8)Mean no. of spikes at preferred directions for ON-OFF DSRGCs	155	26	14	35	12
* *(std)	(10)	(4)	(3)	(5)
(9)Mean no. of spikes at preferred directions for P-like spike trains	20	14	10	57	12
* *(std)	(4)	(4)	(2)	(6)
(10) Mean no. of spikes at preferred directions for ONDS	56	19	12	41	3
* *(std)	(8)	(4)	(2)	(6)
(11) Mean DSi for P-like spike trains	0.76	0.76	0.73	0.53	12
* *(std)	(0.14)	(0.13)	(0.14)	(0.14)

Each number indicates the mean value. Standard deviation is presented within the brackets. Second column represents the description of the quantified parameter, next four columns show the values for each of the ISI category, and the last column represents the number of the recorded cells.

**Table 2:** Summary of information rates results for all cells.

	0 < ISI < 10 (ms)	10 < ISI < 20 (ms)	20 < ISI < 50 (ms)	50 < ISI < 100 (ms)	No. of cells
(1) Entropy for ON-OFF DSRGCs	2.03	1.26	0.85	0.55	12
(std) (bits/spikes)	(0.49)	(0.52)	(0.5)	(0.4)
(2) DSi Entropy for ON-OFF DSRGCs	0.54	0.45	0.40	0.35	12
(std)	(0.17)	(0.2)	(0.2)	(0.2)
(3) DSi Entropy for all cells using white noise stimulus	0.7	0.41	0.32	0.15	20
(std)	(0.2)	(0.2)	(0.2)	(0.1)
(4) Mean ISI at preferred directions	28	29	43	26.6	3
(std) ONDS (percentage of total ISI %)	(4)	(0.6)	(13)	(2)
(5) Mean ISI at opposed directions	2	1	2.1	5.1	3
(std) ONDS (percentage of total ISI %)	(0.4)	(0.6)	(1)	(0.8)
(6) Mean DSi for ONDS	0.37	0.34	0.52	0.36	3
(std)	(0.1)	(0.07)	(0.08)	(0.03)
(7) Mean DSi Entropy for ONDS	0.32	0.25	0.49	0.55	3
(std)	(0.1)	(0.1)	(0.1)	(0.07)
(8) Mean Entropy for ONDS	0.52	0.39	0.51	0.25	3
(std) (bits/spikes)	(0.1)	(0.1)	(0.3)	(0.19)

Each number indicates the mean value. Standard deviation is presented within the brackets. Second column represents the description of the quantified parameter, next four columns show the values for each of the ISI category, and the last column represents the number of the recorded cells.
